# Comment on “The Biomedical Publications Industry Must Change to Better Serve the Needs of Science and Scientists”

**DOI:** 10.20411/pai.v10i2.833

**Published:** 2025-06-12

**Authors:** Abeera Saleem Mughal, Hafiz Shahbaz Zahoor

**Affiliations:** 1 Jinnah Sindh Medical University, Karachi, Pakistan; 2 Quaid-e-Azam Medical College, Bahawalpur, Pakistan

## DEAR EDITOR-IN-CHIEF,

The paper titled “The Biomedical Publications Industry Must Change to Better Serve the Needs of Science and Scientists” [1] raises an important and timely issue. I appreciate the authors’ efforts in highlighting challenges that strike at the core of scientific progress. As a researcher myself, I could deeply relate to many of the issues described. However, I believe a few important aspects could have been explored further.

While the journal’s effort to evaluate peer reviews is a step in the right direction, relying solely on internal ratings by senior editors can introduce bias. The lack of transparency often makes the system operate like a black box, with limited checks on editorial influence. Introducing greater openness would add an additional layer of quality assurance and help build trust in the system [2].

Although the commentary rightly highlights the burden of high article processing charges (APCs), it’s also important to ask: who gets to publish, and who gets left out? The current system quietly excludes researchers from low- and middle-income countries (LMICs) who are unable to share their work because they simply can’t afford to. With APCs averaging around 2,000 USD in journals published by BMC, MDPI, and Sage Publications, an amount equivalent to several months’ salary for a principal investigator, this becomes a serious barrier. Although fee waivers exist, they are often hard to get and seem more like a procedural obstacle than sincere assistance [3].

Finally, while the authors suggest their proposed policies can be implemented at minimal or no cost, the commentary ends without offering a roadmap for practical application. This renders the message incomplete. Undoubtedly *Pathogens and Immunity* is doing commendable work, but one journal alone cannot shift the tide. To truly support researchers globally, a system-wide change is necessary. If the authors could outline practical steps—such as standardizing fee waiver criteria across journals and promoting open peer review practices or encouraging consortia to subsidize APCs—it would pave the way for real, lasting change.
